# Administration of Hookworm Excretory/Secretory Proteins Improves Glucose Tolerance in a Mouse Model of Type 2 Diabetes

**DOI:** 10.3390/biom12050637

**Published:** 2022-04-26

**Authors:** Zainab Khudhair, Rafid Alhallaf, Ramon M. Eichenberger, Matt Field, Lutz Krause, Javier Sotillo, Alex Loukas

**Affiliations:** 1 Centre for Molecular Therapeutics, Australian Institute of Tropical Health and Medicine, James Cook University, Cairns, QLD 4878, Australia; zainab.agha@my.jcu.edu.au (Z.K.); rafid.alhallaf@my.jcu.edu.au (R.A.); ramon.eichenberger@uzh.ch (R.M.E.); matt.field@jcu.edu.au (M.F.); javier.sotillo@isciii.es (J.S.); 2 Department of Neurology, Johns Hopkins University School of Medicine, Baltimore, MD 21211, USA; 3 Institute of Parasitology, Vetsuisse Faculty, University of Zurich, CH-8057 Zurich, Switzerland; 4 John Curtin School of Medical Research, Australian National University, Canberra, ACT 2601, Australia; 5 Microba Life Sciences, Brisbane, QLD 4000, Australia; lutz.krause@microba.com; 6 Parasitology Reference and Research Laboratory, Centro Nacional de Microbiología, Instituto de Salud Carlos III, Majadahonda, 28220 Madrid, Spain

**Keywords:** Nippostrongylus, helminth, diabetes, excretory/secretory products, T helper 2, eosinophils, M2 macrophage

## Abstract

Diabetes is recognised as the world’s fastest growing chronic condition globally. Helminth infections have been shown to be associated with a lower prevalence of type 2 diabetes (T2D), in part due to their ability to induce a type 2 immune response. Therefore, to understand the molecular mechanisms that underlie the development of T2D-induced insulin resistance, we treated mice fed on normal or diabetes-promoting diets with excretory/secretory products (ES) from the gastrointestinal helminth *Nippostrongylus brasiliensis*. We demonstrated that treatment with crude ES products from adult worms (AES) or infective third-stage larvae (L3ES) from *N. brasiliensis* improved glucose tolerance and attenuated body weight gain in mice fed on a high glycaemic index diet. *N. brasiliensis* ES administration to mice was associated with a type 2 immune response measured by increased eosinophils and IL-5 in peripheral tissues but not IL-4, and with a decrease in the level of IL-6 in adipose tissue and corresponding increase in IL-6 levels in the liver. Moreover, treatment with AES or L3ES was associated with significant changes in the community composition of the gut microbiota at the phylum and order levels. These data highlight a role for *N. brasiliensis* ES in modulating the immune response associated with T2D, and suggest that *N. brasiliensis* ES contain molecules with therapeutic potential for treating metabolic syndrome and T2D.

## 1. Introduction

Type 2 diabetes (T2D) is a metabolic disease resulting from defects in protein, fat and carbohydrate metabolism and elevated levels of glucose in the blood that lead to impairment in insulin action and to relative deficiency in insulin secretion [1]. The incidence of T2D is increasing in all regions of the world. According to the International Diabetes Federation, there were 424.9 million people with diabetes in 2017 and this number is expected to reach 628.6 million people by 2045, with T2D accounting for 90% of these cases [1].

It is well established that helminth infection induces T helper type 2 (Th2) and regulatory T cell (Treg) immune responses in infected hosts as a survival strategy for these chronic pathogens [2]. The resulting immune response suppresses immunopathology induced by infection with these large parasites but also contributes to overall protection against immune mediated diseases [3]. It is also becoming apparent that helminth secretions or excretory/secretory (ES) products include potent factors that can modulate the host’s immune response, contributing to the control or prevention of inflammation-mediated diseases [4]. The adult developmental stage of the human hookworm, *Necator americanus* secretes at least 200 detectable proteins when cultured in vitro [5], and ES products released by related hookworms that infect animals have been shown to possess potent immunoregulatory properties characterised by a polarisation towards the production of Tregs, type-2 innate lymphoid cells (ILC2s), tolerogenic dendritic cells (DCs), M2 macrophages (MACS), and anti-inflammatory cytokines such as IL-10 and TGFβ [6,7]. Indeed, it has been previously reported that crude ES products of the adult canine hookworm, *Ancylostoma caninum* (*Ac*ES)—including a recombinant version of the most abundant *Ac*ES protein, AIP-1—and the low molecular weight metabolites found in *Ac*ES were all capable of protecting against chemically-induced colitis in mice [8,9,10,11]. Protection against gastrointestinal inflammation was associated with induction of Th2/anti-inflammatory cytokines such as IL-4, IL-5, IL-10 [8], TGF-β and thymic stromal lymphopoietin (TSLP) [9], and downregulation of Th1/pro-inflammatory cytokines such as IL-6, IL-17, IFN-γ [8], and TNF-α [9], as well as recruitment of M2 MACs, eosinophils [8] and Tregs [9] to the site of ES administration [8,9]. It has also been shown that the recombinant protein *Ac-*AIP-2, one of the most abundant *Ac*ES proteins, suppressed airway inflammation in a mouse model of asthma via induction of tolerogenic DCs and FoxP3^+^ Tregs [12]. ES products of another GI nematode, *H. polygyrus* (HES) also impaired DC function and induced Tregs [13,14]. ES products of the rat hookworm *Nippostrongylus brasiliensis* have been found to modulate DCs, favouring the induction of Th2 immune responses [15]. Moreover, in a recent study, extracellular vesicles from the ES products of *N. brasiliensis* were shown to protect against chemically-induced colitis in mice. This was associated with suppression of the inflammatory cytokines IL-6, IL-1β, IFNγ and IL-17a and an increase in the anti-inflammatory cytokine IL-10 [16]. Furthermore, ES products from adult *N. brasiliensis* [17], and recombinant forms of the cysteine protease inhibitors rNB-Cys from *N. brasiliensis* and rHp-CPI from *H. polygyrus* suppressed antigen-specific antibody production [18] and IFNγ production [19], and simultaneously induced ES-specific Th2 immune responses [17] in ovalbumin-immunised mice [17,18,19].

In the context of diabetes, the ES products from parasitic platyhelminths (flatworms) have been shown to confer protection against T1D and T2D. For instance, the ES products of the liver fluke *Fasciola hepatica*, and soluble egg antigen (SEA) from the blood fluke *Schistosoma mansoni* prevented T1D in NOD mice via induction of regulatory B cells, Tregs, M2 MACs and DCs, and increased IL-4, IL-10 and TGF-β levels [20,21]. Moreover, treatment with *S. mansoni* SEA [22], LNFPIII glycan from SEA [23], and the recombinant SEA T2 RNase ω1 [24] improved insulin sensitivity and suppressed liver lipogenesis in diabetic obese mice fed a high fat diet. This was associated with increased numbers of ILC2s, eosinophils and M2 MACs and increased levels of Th2 cytokines and the alarmin IL-33 in adipose tissue and liver [22,23,24,25]. Administration of *Schistosoma japonicum* SEA also protects mice against T2D via induction of Tregs and Th2 immune responses, characterised by an increase in the frequency of CD25^+^Foxp3^+^ T cells, and elevated expression of IL-4 and IL-5 in the spleen [26].

In terms of nematode roundworm parasites, soluble somatic products from the whip worm *Trichuris suis* [25] and the filarial nematode *Litomosoides sigmodontis* improved metabolic homeostasis and insulin sensitivity in a murine obesity model, and the latter was associated with increased numbers of eosinophils, M2 MACs, and ILC2s [27]. To our knowledge, however, ES products from parasitic nematodes have not yet been shown to confer either prophylactic or therapeutic protection against metabolic syndrome, although when hookworm ES products are heat denatured and enzymatically digested, they lose their ability to drive Th2 cytokine production and protect against inducible colitis in mice [8].

A growing body of evidence suggests that helminth infections (both flatworms and roundworms) can alter the gut microbial community, leading to protection against immune-mediated diseases such as allergic inflammation, colitis, obesity, arthritis, and lupus [28,29,30,31,32,33,34]. Therefore, in this study, we investigated the effect of nematode ES products in modulating the host’s immune system and the gut microbiota composition in mice fed a diabetogenic diet. We describe a role for ES products of *N. brasiliensis* in modulating the immune response associated with T2D. Intraperitoneal injection of ES products resulted in reduced glucose levels and reduced body weight gain in a mouse model of T2D, and was accompanied by activation of eosinophilia and elevated IL-5, as well as a shift in the microbiota profile between the groups treated with ES products compared with untreated littermates fed both normal chow and high glycaemic index diets. Together, these data highlight, for the first time, the importance of gastrointestinal nematode ES products in modulating the immune response associated with T2D, and imply that recombinant forms of parasitic nematode ES proteins could be developed as therapeutics for metabolic syndrome.

## 2. Materials and Methods

### 2.1. Ethics Statement

All procedures were approved by the James Cook University Animal Ethics Committee, ethics application number A2244. The study protocols were in accordance with the 2007 Australian Code of Practice for the Care and Use of Animals for Scientific Purposes and the 2001 Queensland Animal Care and Protection Act. *N. brasiliensis* was maintained in Sprague–Dawley rats (Animal Resources Centre, Perth, WA, Australia) as described elsewhere [35] (ethics application number A2300). The *N. brasiliensis* life cycle was maintained in Sprague–Dawley rats (Animal Resources Centre, Perth, WA, Australia) in our lab (ethics application number A2300) [36].

### 2.2. Animals and Diet

Male C57BL/6 wild-type (WT) mice at 5 weeks of age were separated into 2 main groups: one group was fed a normal chow (NC) diet and the other group was fed a high glycaemic index (HGI) diet, with a glycaemic index of close to 100 (SF03-30; Speciality Feeds, Western Australia) to induce T2D (Supplemental Table S1). Food in each cage was weighed weekly.

### 2.3. ES Preparation and Administration

Briefly, faeces from *N. brasiliensis*-infected rats were collected from days 5–9 post-infection. Egg-containing faeces were mixed with an equal amount of water and charcoal, distributed into Petri dishes and incubated at 26 °C. One week after incubation, infective third-stage larvae (L3) were collected from the faecal/charcoal culture plate, washed three times with PBS, then three times with PBS supplemented with 5% antibiotic-antimycotic (AA) (Gibco). L3 were then transferred into a flat bottom 24-well plate, cultured in serum-free RPMI media (Gibco) supplemented with 2% AA and 1% D-glucose (Sigma, St. Louis, MO, USA) and incubated at 37 °C and 5% CO_2_ at a density of 500 L3/well. The supernatant was then collected daily from days 1–10, ensuring motility of the worms at all times. Supernatants were stored at −30 °C before protein concentration was determined. This supernatant contained the *N. brasiliensis* L3 excretory/secretory products (L3ES), the protein constituents of which have been previously reported [36].

For adult stage ES (AES) product preparation, rats were infected with 3000 L3 *N. brasiliensis*. At day 6, post-infection rats were euthanised, and the small intestines were collected and opened in a Petri dish containing RPMI and incubated at 37 °C with 5% CO_2_ for 2 h. Adult worms were then collected, washed three times with PBS followed by three washes with PBS supplemented with 5% AA and one wash with RPMI media supplemented with 2% AA. One hundred (100) worms/well were then transferred into a flat bottom 24-well plate and incubated in RPMI media supplemented with 2% AA, 1% D-glucose and 1% Glutamax at 37 °C and 5% CO_2_. After 24 h, the supernatant was discarded to minimise contamination with host proteins and replaced with new media. AES was collected at day 2 and replaced subsequently every day until day 10 with fresh media. Supernatants were stored at −30 °C before protein concentration was determined.

Amicon ultrafiltration 3 kDa centrifugal concentrators (Thermo scientific, Waltham, MA, USA) were used for protein concentration and buffer exchanged with PBS. The Pierce BCA protein assay kit (Thermo scientific) was used for quantification of the protein content following the manufacturer’s instructions. The Pierce LAL Chromogenic Endotoxin Quantification Kit (Thermo Scientific) was used for the quantification of lipopolysaccharide (LPS) in AES and L3ES preps. Firstly, AES and the L3ES were incubated with 1% Triton-114 at 4 °C on a rotor (Ratek) overnight with low constant stirring. After that, ES proteins were incubated at 37 °C for 10 min using a dry bath incubator (Major Science), followed by centrifugation in a Microfuge 22R centrifuge (Beckman Coulter) at 14,000× *g* for 5 min. The top layer was collected and prepared for LPS quantification. Briefly, 4 standards were prepared, ranging from 1- 0.1 EU/mL. Then, 50 μL of the standards and ES samples were added in duplicate to a 96-well plate and incubated at 37 °C and 5% CO_2_ for 5 min. Subsequently, 50 μL of Limulus Amebocyte Lysate was added and the plate was incubated at 37 °C for 10 min, followed by addition of 50 μL of Chromogenic Substrate and incubation for 6 min at 37 °C. Then, 50 μL of the stop solution was added and the results were read on a BMG Polarstar Omega fluorescence microplate reader at 405 nm. The ES proteins were suitable for use when the LPS content was below 5 EU/mg of protein (https://www.fda.gov/inspections-compliance-enforcement-and-criminal-investigations/inspection-technical-guides/bacterial-endotoxinspyrogens (accessed on 12 April 2022)).

The ES products were administered to mice intraperitoneally twice weekly from week 6 until the end of the experiment at a dose of 1 mg/kg. We have shown that hookworm ES products at 1 mg/kg is therapeutic/prophylactic for a range of inflammatory conditions in mice, including colitis [8] and asthma [12]. Control groups were administered intraperitoneally with the same volume of PBS. Group sizes were n = 5 and the experiment was conducted twice. Data from both experiments were pooled for presentation.

### 2.4. Fasting Blood Glucose and Oral Glucose Tolerance Test

Food was withdrawn for 6 h prior to measuring fasting blood glucose (FBG) in mice. Blood sampling was performed by tail bleeding using Accu-Check^®^ Performa (Roche). Mice were considered diabetic when glucose levels reached >12.0 mmol/L. The oral glucose tolerance test (OGTT) was measured in 6 h unfed mice after initial blood collection (time 0). Mice were administered D-glucose orally (2 g/kg body weight) by gavage. Blood sampling was performed by tail bleeding at 15, 30, 60, 90, and 120 min after administration of glucose.

### 2.5. Tissue Collection and Cytokine Analysis

Adipose tissue (AT), liver, and small intestine were collected and washed with PBS; one cm of the tissue was combined with 0.5 mL of PBS, homogenised using a TissueLyzer (QIAGEN), centrifuged, and supernatants stored at −80 °C until use. The ELISA Ready-Set-Go set (eBioscience) for the cytokines IL-4, IL-5, and IL-6 was used following the manufacturer’s instructions. In brief, a 96-well flat bottom plate was coated with 50 μL of capture antibody and incubated overnight at 4 °C. The plate was then washed 3 times with washing buffer. Then, 50 μL of blocking reagent was added and the plate was incubated for 1 h at room temperature. The washing step was performed twice. Fifty (50) μL from the samples and from serial dilutions of the standards were added to the plate and incubated overnight at 4 °C, followed by 3 more washing steps. Subsequently, 50 μL of detection antibody was added, incubated for 30 min at room temperature and followed by 3 more washes. Then, 50 μL of substrate solution was added and incubated for 15 min at room temperature. Finally, 50 μL of stop solution (1M HCl) was added and the absorbance was read on a BMG Polarstar Omega plate reader at 450 nm. Mesenteric lymph nodes (mLN), epididymal fat pads or liver from male mice fed a NC or HGI diet were removed, as previously described [37].

### 2.6. Flow Cytometry

Cell surface markers were stained for 30 min at 4 °C with rat anti-mouse CD3/CD19-CF594 (Clone:145-2C11,1D3) F4/80-APC (Clone: T45-2342), CD11c-FITC (Clone: HL3), CD301-pecy7 (Clone: LOM-14), CD64-PerCp-Cy5.5 (Clone: X45-5/7.1), CD11b-BV650 (Clone: M1/70), Ly6G-efluor700 (Clone: 1A8), and Siglec-F-PE (Clone: E50-2440) (BD Bioscience). All antibody incubations were performed at 4 °C for 30 min (isotype controls were included). Data were acquired using a BD FACS Aria with BD FACS DIVA software (BD Bioscience) and analysed using FlowJo software (Tree Star, Inc., Ashland, OR, USA).

### 2.7. Data Analysis

Data were tested for statistical significance using GraphPad Prism software (version 8). The Mann-Whitney U test was applied to test differences between two unpaired groups with nonparametric distribution for statistical significance. Data that were normally distributed were tested for statistical significance using the unpaired *t*-text for comparisons of two groups or the ANOVA test followed by the Holm-Sidack multiple-comparison test to compare more than two groups. Values of *p* < 0.05 were considered statistically significant. Results are expressed as means ± SEM. * *p* < 0.05; ** *p* < 0.01; *** *p* <0.001.

### 2.8. DNA Extraction and Bacterial 16S rRNA Illumina Sequencing

Faecal samples were collected and stored immediately at −80 °C for further analysis. DNA extraction and 16 s rRNA sequencing were performed by the Australian Centre for Ecogenomics, University of Queensland, Brisbane as previously described [37].

### 2.9. Bioinformatics and Statistical Analysis

Microbiome differential analysis was performed using a modified version of MetaDEGalaxy [38]. In short, raw reads were run through fastqc for quality control, Trimmomatic [39] for adapter trimming and low quality base removal, QIIME [40] for Operational Taxonomic Units (OTUs) generation, and BLAST [41] for OTU identification. Within QIIME, low-quality reads are filtered with all remaining sequences de-multiplexed and chimeric sequences removed using UCHIME [42]. Sequences were subsequently clustered into OTUs on the basis of similarity to known bacterial sequences in the Greengenes database [43] (cut-off: 97% sequence similarity) using the UCLUST software [44].

For each biom file, the taxonomic observation and metadata were added using biom API [45] which was next loaded into the R package phyloseq [46]. Within phyloseq, the DESeq2 [47] API was called and a list of most differentially expressed bacteria generated for all possible pairings of conditions (NC and NC L3ES or NC AES, HGI and HGI L3ES or HGI AES). All subsequent plots were generated using ggplot2 and Calypso online software (version 8.84) (http://cgenome.net/calypso/ (accessed on 17 July 2020)) [48]. Within Calypso, data were normalised by total sum normalisation (TSS) combined with square root transformation. Multivariate redundancy analysis to overall differences in the microbial profile between groups and Adonis based on the Bray-Curtis dissimilarity were used. Differences in bacterial alpha diversity (Shannon diversity) and richness between groups were used. Values of *p* < 0.05 were considered statistically significant following false discovery rate (FDR) correction. Differences in the bacterial taxa abundance between groups were assessed using ANOVA-like differential expression analysis (ALDEx2) and quantitative visualisation of phyla abundance.

## 3. Results

### 3.1. Treatment with Either AES or L3ES from N. brasiliensis Improves Glucose Tolerance and Attenuates Body Weight Gain

**Figure 1:**
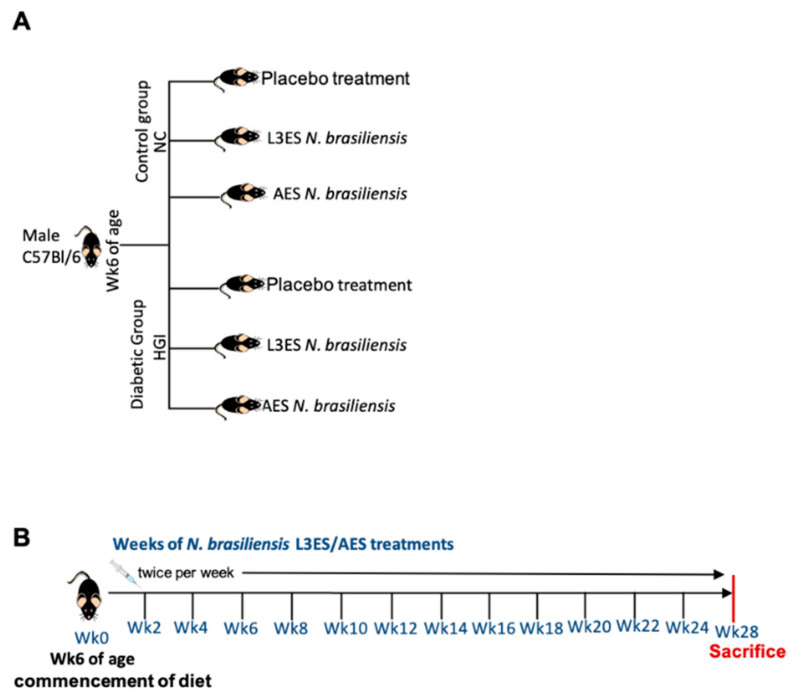
Experimental design and timeline for treatments of mice with *Nippostrongylus brasiliensis* third stage larvae (L3) or adult worm (**A**) excretory secretory proteins (ES). Six-week-old C57BL/6 mice were fed normal control (NC) or high glycaemic index (HGI) diets (**A**) and injected intra-peritoneally twice weekly with 1 mg/kg of L3ES or AES commencing at week 0 of the experiment and ending with sacrifice of the animals and tissue collection at week 28 (**B**). Food in each cage was weighed weekly and no differences were detected between diets or treated vs. untreated mice.

In order to address the prophylactic effect of *N. brasiliensis* ES on the outcome of T2D, five-week-old male C57BL/6 mice were used; half the mice were fed NC diet and the other half were fed an HGI diet to induce T2D for up to 30 weeks (Figure 1A). Mice were treated twice weekly with *N. brasiliensis* AES or L3ES at a dose of 1 mg/kg starting at week 6 until the end of the experiment (Figure 1B).

**Figure 2:**
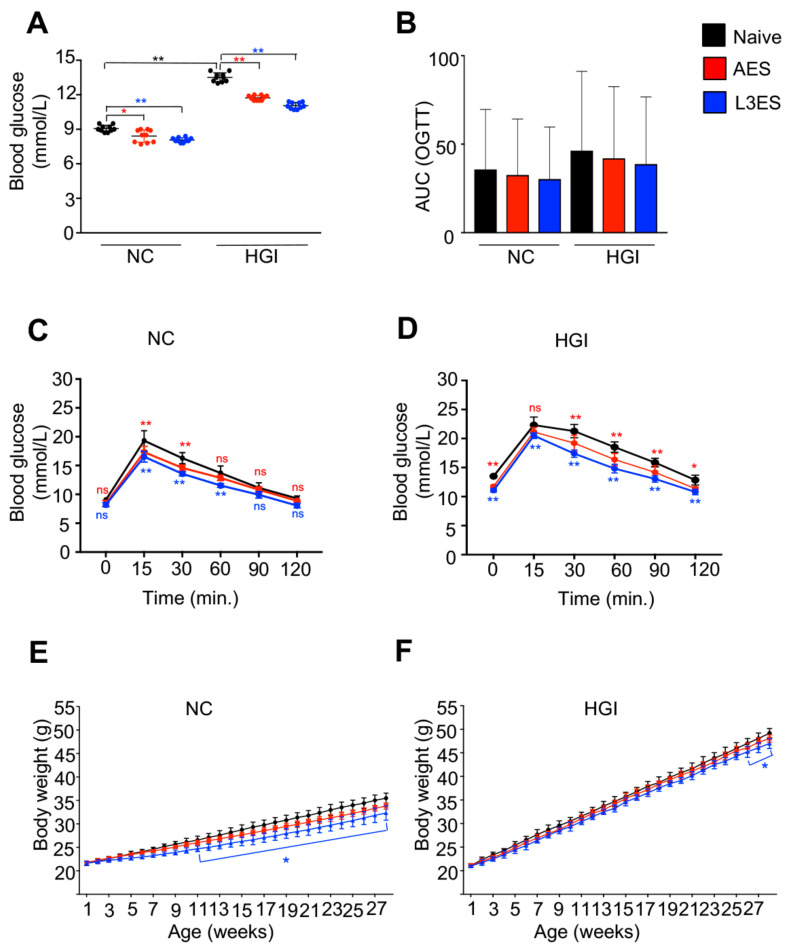
Treatment of six-week-old mice with excretory/secretory (ES) products derived from *Nippostrongylus brasiliensis* third-stage larvae (L3ES) or adult worms (AES) resulted in decreased fasting blood glucose (FBG), improved glucose metabolism and reduced weight gain in a high glycaemic index (HGI) model of type 2 diabetes. (**A**) FBG in mice fed on normal control (NC) diet or HGI diet and administered L3ES or AES. (**B**) Area under the curve (AUC) for oral glucose tolerance test (OGTT) data in mice fed on NC or HGI diets and administered L3ES or AES; (**C**) Blood glucose levels in mice fed an NC diet (**C**) or a HGI diet (**D**) and administered L3ES or AES. Body weight of mice fed an NC diet (**E**) or a HGI diet (**F**) and administered L3ES or AES. Statistical significance was determined with Student’s *t*-text or two-way analysis of variance (ANOVA). Data are expressed as means ± SD from 2 pooled experiments where n = 5/group for each experiment. Naïve vs. AES ^*^ (red asterisk), Naïve vs. L3ES ^*^ (blue asterisk), Naïve NC diet vs. Naïve HGI diet (black asterisk); * *p* < 0.05; ** *p* < 0.01. ns = not significant, *p* > 0.05.

Two days prior to sacrifice, FBG and OGTT were measured. As predicted, mice on HGI diet had significantly higher levels of FBG compared to those on an NC diet (Figure 2A). Mice on a HGI diet treated with AES or L3ES showed a significant decrease in FBG levels compared to the untreated control group (Figure 2A). Moreover, mice on an NC diet treated with AES or L3ES showed a significant decrease in FBG levels compared to the diet-matched untreated control group (Figure 2A). Furthermore, results from the OGTT test showed that HGI diet-fed mice treated with AES or L3ES had significantly lower levels of blood glucose compared to the untreated diet-matched control group (Figure 2D). The OGTT test in NC diet groups treated with AES or L3ES also revealed significant decreases in the levels of blood glucose compared to the untreated diet-matched control group (Figure 2C). Reduction in body weight gain was also observed as a result of the treatment. Mice fed either NC or HGI diets and treated with AES or L3ES gained less weight compared to their diet-matched untreated control groups (Figure 2E,F).

### 3.2. Treatment with N. brasiliensis ES Induces Tissue Eosinophilia and Th2 Immune Responses

**Figure 3:**
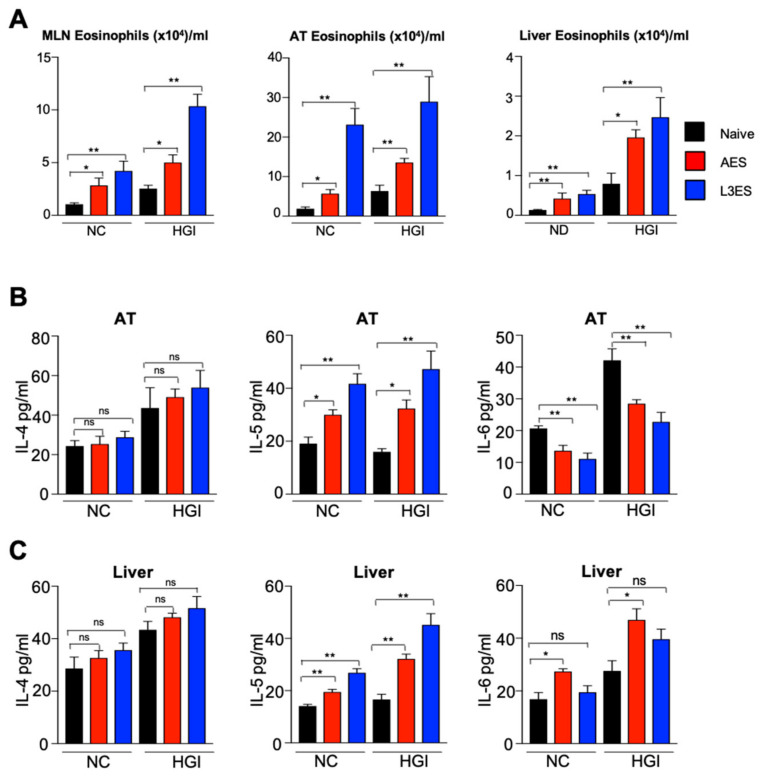
Increase in the frequency of eosinophils in the mesenteric lymph nodes (MLN), adipose tissue (AT), and liver in mice fed a normal control (NC) or high glycaemic index (HGI) diet and treated with excretory/secretory (ES) products from *Nippostrongylus brasiliensis* third-stage larvae (L3ES) or adult worms (AES): (**A**) Eosinophil frequency and total numbers in mLNs, AT, and liver; (**B**) AT IL-4, IL-5 and IL-6 expression; (**C**) Liver IL-4, IL-5, and IL-6 expression. Statistical significance was determined with Student’s *t*-text. Data are expressed as means ± SEM from 2 pooled experiments where n = 5/group for each experiment. * *p* < 0.05; ** *p* < 0.01. ns = not significant, *p* > 0.05.

At termination, mLN, AT and liver were collected from mice for characterisation of the Th2 immune response. In response to *N. brasiliensis* AES or L3ES injection, there was a significant increase in the total number of eosinophils in the mLN of the HGI diet groups compared to their respective naïve groups (Figure 3A). Moreover, NC diet groups treated with *N. brasiliensis* AES or L3ES had significant increases in the total number of eosinophils in the mLN, AT and liver compared to the untreated control group (Figure 3A). Moreover, there was a significant increase in the total number of eosinophils in the AT of HGI diet groups treated with L3ES or AES, as well as the AT of NC diet groups treated with *N. brasiliensis* AES or L3ES. Furthermore, the total number of eosinophils was significantly higher in the liver of HGI diet groups treated with L3ES and AES or in the NC diet groups treated with *N. brasiliensis* AES and L3ES compared to their respective untreated groups (Figure 3A).

To assess whether the increase in eosinophil numbers and associated Th2 response was associated with elevated Th2 cytokine levels in AT and liver, we analysed cytokine responses by ELISA. Treatment with AES or L3ES increased the levels of IL-4 and IL-5 in AT and liver (Figure 3B,C). Both HGI and NC diet groups treated with AES or L3ES had significantly higher levels of AT IL-5 compared to their respective untreated control groups (Figure 3B). Moreover, IL-4 and IL-5 levels were significantly higher in the liver of both HGI diet groups and NC diet groups treated with AES or L3ES compared to their untreated control groups (Figure 3C). We also found a trend towards increased levels of IL-4 in the AT of AES- and L3ES-treated mice on both diets compared to their respective untreated control groups (Figure 3B), although this did not reach significance (Figure 3B,C). Moreover, mice treated with either AES or L3ES on both NC and HGI diets had significantly lower levels of IL-6 in the AT (Figure 3B) compared to untreated diet-matched controls. Of note, however, mice treated with AES but not L3ES on both diets displayed a significant increase in the level of IL-6 in the liver compared to their untreated diet-matched control groups (Figure 3C).

### 3.3. Treatment with N. brasiliensis ES Products Affects Microbial Composition of Mice Fed on Normal and Diabetogenic Diets

**Figure 4:**
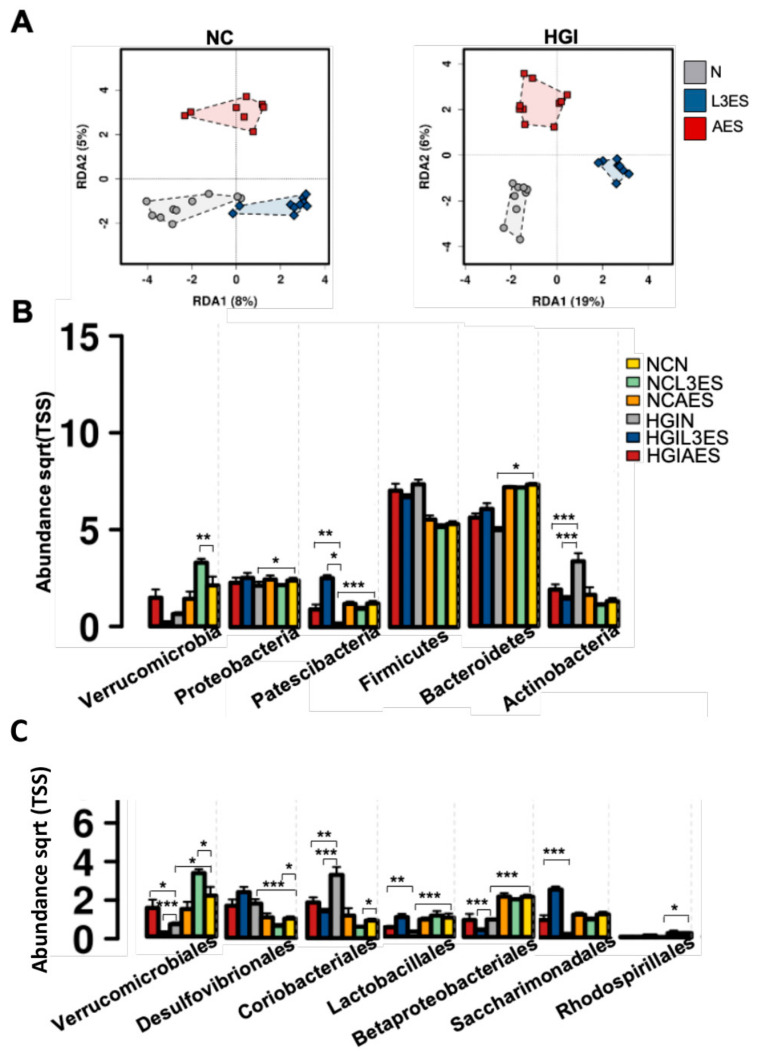
Multivariate analysis of differences in the microbial profiles in the small intestine of mice fed a normal control (NC) or high glycaemic index (HGI) diet and treated with excretory/secretory (ES) products from *Nippostrongylus brasiliensis* third-stage larvae (L3ES) or adult worms (AES): (**A**) Relative abundance of bacterial phyla in the small intestine of mice fed on NC or HGI diets and treated with L3ES or AES (**B**); Relative abundance of defined bacterial orders in the small intestine of mice fed a NC or HGI diet and treated with L3ES or AES (**C**). *p*-values are based on ANOVA-like differential expression analysis and are from 2 pooled experiments where n = 5/group for each experiment. * *p* < 0.05, ** *p* < 0.01, *** *p* < 0.001.

In order to address the effect of treatment with L3ES or AES of *N. brasiliensis* on the composition of gut microbiota, stool samples were collected at termination. RDA at the OTU level showed significantly different microbial compositions at the phylum level in both NC and HGI diet groups treated with L3ES or AES compared to their untreated control littermates (Figure 4A,B). The AES, but not the L3ES-treated group, fed an NC diet showed significant differences in microbial diversity compared to untreated, diet-matched littermates (Table 1). Adonis analysis based on Bray-Curtis and Spearman index showed significant differences between L3ES- and AES-treated groups on an HGI diet compared to the untreated diet-matched control group based on Bray-Curtis and Spearman index (Table 1). The α-diversity increased significantly in AES- but not L3ES- treated mice on an NC diet compared to the untreated diet-matched control group (Supplementary Figure S1A). Differences in species richness were not statistically significant between the NC diet groups treated with L3ES or AES (Supplementary Figure S1A). The α-diversity and the species richness differences increased significantly in the HGI diet group treated with L3ES (but not AES) compared to their untreated control group (Supplementary Figure S1B).

### 3.4. Treatment with L3ES or AES Alters the Abundance of Bacterial Taxa

Untreated groups fed on both NC and HGI diets displayed differences in abundance of bacterial phyla. The untreated NC diet group (NCN) showed significantly higher abundance of Bacteroidetes, Proteobacteria and Patescibacteria phyla compared to the untreated HGI diet group (HGIN) (Figure 4B). Although the NC diet group treated with L3ES or AES (NCL3ES or NCAES) showed no difference in the abundance of the latter phyla compared to the NCN group, the HGI diet groups treated with L3ES or AES (HGIL3ES or HGIAES) displayed a trend towards increased abundance of Bacteroidetes phylum and had significantly higher abundance of Patescibacteria phylum compared to the HGIN group (Figure 4B). The Verrucomicrobia abundance was significantly increased in the NCL3ES group but not in the NCAES group compared to the NCN group, but this was not replicated in mice fed the HGI diet (Figure 4B). Actinobacteria phylum displayed an increased abundance in the HGIN group compared to the NCN group. Interestingly, the HGIL3ES and HGIAES groups exhibited a significant decrease in the abundance of Actinobacteria phylum compared to the HGIN group, however no such differences were observed between treatment groups on the NC diet (Figure 4B). At the order level, abundance of Lactobacillales was significantly decreased, and abundance of Saccharimonadales displayed a trend towards a decrease in the HGIN group compared to the NCN group. Of note, the abundance of Lactobacillales and Saccharimonadales showed a significant increase in the HGIAES group, and a non-significant trend towards an increase in the HGIL3ES group compared to the HGIN group (Figure 4C). On the other hand, Coriobacteriales abundance was significantly reduced in the HGIL3ES and HGIAES groups compared to the HGIN group (Figure 4C). Abundance of Verrucomicrobiales and Betaproteobacteriales was significantly decreased, while abundance of Desulfovibrionales was significantly increased in the HGIN group compared to their respective NCN group (Figure 4C). On the other hand, abundance of Verrucomicrobiales significantly increased in the NCL3ES group as well as in the HGIAES group compared to their respective untreated groups (Figure 4C). However, the latter group showed significant decrease in the HGIL3ES group, as well as in the NCAES group compared to their respective untreated groups (Figure 4C). Betaproteobacteriales’ abundance significantly decreased in HGIL3ES (but not NCL3ES, NCAES or HGIAES groups) compared to their untreated naïve group (Figure 4C). Abundance of Desulfovibrionales significantly decreased in the NCL3ES group, but this was not replicated in mice fed the HGI diet (Figure 4C). Abundance of Rhodospirillales showed significant decrease in the NCAES group (but not NCL3ES, HGIL3ES or HGIAES groups) compared to their untreated naïve group (Figure 4C).

## 4. Discussion

Incidence and prevalence of diabetes is on the rise globally, imposing a drastic socio-economic burden on public health [1]. Many researchers have reported that helminths and their secretions can modulate the host’s immune response, which might have beneficial effects in controlling or preventing inflammatory responses associated with immune-mediated diseases [4]. However, evidence for pre-clinical efficacy of helminth ES products in T2D animal models is sparse, and this is the first report from a parasitic nematode. Understanding the mechanisms by which helminth ES products can skew the immune response away from a T2D-promoting phenotype is critical for the rational design of preventative or curative therapies against this disease.

In the present study, we showed that administration of *N. brasiliensis* crude ES from adult and L3 developmental stages attenuated the clinical indicators of T2D in the HGI diet mouse model. This diet contains moderate levels of fat and has a high glycaemic index, and is closely aligned with modern society dietary patterns to better reflect the etiology of T2D [49]. Our data showed that AES and L3ES administration ameliorates glucose intolerance and attenuates body weight gain in the treated groups. Previous findings using SEA from *S. japonicum* significantly reduced the level of blood sugar, however the level was still higher than those on a normal diet [26]. This was consistent with our results, as we also found that treatment with AES or L3ES of *N. brasiliensis* significantly reduced the blood glucose level in the diabetic group, although it was still higher than that of mice fed a normal chow diet.

Our results showed non-significant differences in the levels of IL-4 in the liver and AT of treated groups compared to their naïve counterparts; however, we found a significant increase in the number of eosinophils in the MLN, AT and liver, and a significant increase in the level of IL-5 in the AT and liver. IL-5 is responsible for the expansion and survival of eosinophils [50,51,52], and *N. brasiliensis* infection is characterised by high eosinophil production induced by CD4^+^ T cell-derived IL-5 [53]. Importantly, it has been demonstrated that AT eosinophils are highly dependent on IL-5 [54], and eosinophils play an unexpected role in metabolic homeostasis through maintenance of adipose M2 MACs [55]. As with eosinophil deficiency, IL-5 deficiency in mice impairs eosinophil accumulation in AT, and mice develop increased body fat, impaired glucose tolerance and insulin resistance when fed a high fat diet [54]. Moreover, ILC2s have been found to play an important role in sustaining AT eosinophils [54]. Infection with *N. brasiliensis* induces adipose eosinophilia and enhances glucose tolerance in mice fed a high fat diet via activation of AT ILC2s, which induces IL-5 production that leads to accumulation of eosinophils in the AT [54]. Deletion of IL-5 results in the loss of eosinophil activation in AT [54] and impaired glucose tolerance [55]. It has been reported that *N. brasiliensis* infection protects against experimental autoimmune encephalomyelitis (EAE) via increased IL-5 levels and subsequent increases in eosinophils and CD4^+^CD25^+^ Tregs. Moreover, anti-IL-5 or anti-CD25, but not anti-IL-4, can abolish the beneficial effect of the parasitic infection [56]. Furthermore, treatment of mice with *F. hepatica* ES products attenuated EAE independently of IL-4 and IL-10, but via production of IL-33 and IL-5, which promoted accumulation of eosinophils [57].

In our study, treatment with *N. brasiliensis* AES or L3ES significantly decreased the expression of IL-6 in AT. However, in the liver, IL-6 expression was comparable between L3ES treated and untreated groups, and actually increased in AES treated mice. IL-6 is a pleiotropic and multifaceted cytokine that is involved in the induction of insulin resistance and pathogenesis of T2D [58], and secretion of IL-6 by AT resulted in the induction of hepatic insulin resistance [59]. On the other hand, IL-6 is involved in liver regeneration and maintenance of liver tissue homeostasis [60]. Moreover, IL-6 is required for protection against hepatic inflammation and insulin resistance, and lack of IL-6 reversed the effect [61,62]. Short term preincubation with IL-6 of human PBMC stimulated with anti-CD3 and anti-CD28 antibodies led to up-regulation of IL-4 and IL-5. However, the effect on IL-4 production gradually disappeared but the effect on IL-5 became more pronounced with long term preincubation of these cells with IL-6 [63]. Moreover, IL-6 enhanced expansion and survival of antigen-stimulated CD4^+^ T cells and reduced their apoptosis in vivo and in vitro [64]. Furthermore, in the presence of eosinophil-derived neurotoxin (EDN), splenocytes of TLR2+/+ mice immunised with OVA produced IL-5, IL-6, IL-10, and IL-13, whereas in the presence of LPS, they produced IFN-γ [65]. One possibility for the increase in liver IL-6 we observed might be the effect of IL-6 as a survival factor in liver tissue-mediated EDN in activation of TLR2, and expansion and survival of CD4^+^ T cells in the liver.

We found that treatment with *N. brasiliensis* L3ES or AES at a dose of 1 mg/kg twice weekly significantly decreased the level of blood glucose in diabetic mice, although levels were still higher than the control group. We also found a significant increase in the number of eosinophils in the MLN, AT, and liver associated with an increase in the levels of IL-5 in AT and liver but no differences in the levels of IL-4 in the same tissues. Moreover, a decrease in the level of IL-6 was observed in AT whereas it was increased in the liver. It must be pointed out that *N. brasiliensis* ES products induced airway eosinophilia in a dose-dependent manner [66], and moreover, we (data not published) and others [54,55,67] have found a robust increase in the systemic and peripheral eosinophil numbers as well as IL-4 and IL-5 levels after infection with *N. brasiliensis*. Infection with *N. brasiliensis* requires parasite development through different larval stages and migration through different tissues, which may induce distinct immunomodulatory mechanisms that differ from the i.p. administration of ES products. This might explain the differences in the ability of L3ES and AES to suppress T2D. It is therefore worth considering the route of infection and the therapeutic/prophylactic dose of ES proteins in future studies. As previously mentioned, ES products induce a variety of immune responses from innate and adaptive immune cells, and these cells play very different roles in inducing or protecting against metabolic diseases. Understanding the precise immunological mechanism of action that underpins the clinically beneficial effect is now paramount.

In this study, we found that mice fed the NC diet treated with AES or L3ES and those fed the HGI diet treated with AES showed non-significant increases in microbial α-diversity based on the Shannon index, and mice on the HGI diet treated with L3ES had significantly higher α-diversity. The NCL3ES as well as the HGIL3ES or HGIAES had lower abundance of the Actinobacteria/Coriobacteriales group compared to the untreated controls. The abundance of members of the Coriobacteriaceae family was increased in obese children and adolescents compare to their normal weight counterparts [68]. Patients with T2D had increased abundance of Coriobacteriales in comparison with non-diabetic and pre-diabetic groups [69]. Moreover, rats fed a fructose-rich diet had higher Coriobacteriales/Coriobacteriaceae compared to rats fed a control diet [70], however faecal microbiota transplantation from the latter control group to the high fructose diet group resulted in decreased Coriobacteriaceae abundance [70]. Furthermore, Coriobacteriaceae showed a positive association with non-HDL plasma cholesterol and cholesterol absorption suggested that these bacteria could have a negative impact on cholesterol homeostasis through increasing cholesterol absorption [71]. In contrast, NCL3ES and HGIAES had a higher abundance of Verrucomicrobia/Verrucomicrobiales compared to their control groups, however those on the HGIL3ES had lower abundance of Verrucomicrobiales than their control group. Others have reported a decrease in the abundance of Verrucomicrobia in obese individuals and in both prediabetes and T2D subjects compared to their respective healthy control groups [72,73]. However, others have found an increase in the abundance of Verrucomicrobia in db/db mice, a murine model of T2D, and in mice fed a high fat diet [74,75]. Of note, the gut microbiota of healthy Chilean subjects revealed high abundance of the phylum Verrucomicrobia in comparison with subjects from Papua New Guinea and the Matses ethnic group [76]. Verrucomicrobia are mucin degrading bacteria [77] implicated in health and disease. In this sense, systematic contribution of mucin degrading bacteria in gut homeostasis and dysbiosis has yet to be investigated [78].

Abundance of Desulfovibrionales, Betaproteobacteriales, and Rhodospirillales groups belonging to the Proteobacteria was lower in the NCL3ES, HGIL3ES, and NCAES, respectively, compared to their untreated littermates. The gut microbiota of both obese humans and mice showed higher abundance of the family Desulfovibrionaceae compared to their lean counterparts [79,80]. Supporting evidence comes from animal studies, where mice which were fed a diet that was rich in fat, fructose, and cholesterol had a higher abundance of Rhodospirillales compared to the control diet fed group [81]. Moreover, pre-diabetic and diabetic subjects had a higher abundance of Betaproteobacteria compared to non-diabetic subjects [73,82]. Abundance of Saccharimonadales (belonging to Patescibacteria) and Lactobacillales groups was significantly higher in the HGIAES compare to their untreated group. Patescibacteria are anaerobic fermenters, but this newly identified bacterial group has not been well studied and information on their function is still scarce [83]. *Lactobacillus,* on the other hand, has been widely studied, and different *Lactobacillus* probiotic strains have been reported to improve parameters related to T2D [84]. The bacterial composition can vary in the same individual due to variation in the physiology, pH, and O_2_ tension, digesta flow rates in different anatomical regions, as well as between individuals due to variation in genetic factors, environment, and habitual dietary intake [85]. These variations can be factors contributing to the onset of many diseases and may have potential therapeutic implications [86].

Whether the changes we found in this study are due to direct and/or indirect effects of helminth ES products or a consequence of host immune response and gut microbiota changes, and whether these changes are essential to confer protection against T2D are yet to be investigated. To our knowledge, this study is the first to uncover a novel effector response of nematode ES product-mediated improvement of glucose tolerance in a mouse model of T2D, and to indicate that these ES products hold promise as a novel intervention approach for the treatment of metabolic syndrome. Future work should focus on the duration of the treatment effect in both prophylactic and therapeutic settings, as well as defining the bioactive ES components and their recombinant or synthetic production and validation.

## Figures and Tables

**Table 1:** Significant differences in alpha diversity based on Adonis *p*-values determined using Bray-Curtis and Spearman index methods.

Group	*p*-Value Bray-Curtis	*p*-Value Spearman Index
NC-N vs. NC-L3ES	0.01	0.004
NC-N vs. NC-AES	0.14	0.1
HGI-N vs. HGI-L3ES	0.0003	0.0003
HGI-N vs. HGI-AES	0.0006	0.0003

## Data Availability

The data presented in this study are available on request from the corresponding author. The data are not publicly available due to commercial in confidence.

## Supplementary Materials

The following supporting information can be downloaded at https://www.mdpi.com/article/10.3390/biom12050637/s1, Figure S1: Shannon index and richness in the small intestine of mice fed on NC diet or HGI diet and treated with crude *Nippostrongylus brasiliensis* L3ES or AES; *p*-values are based on multiple linear regression and are representative of 1 experiment where n = 10/group. *** *p* < 0.001; Table S1: Composition of diets fed to mice in this study. NC—normal chow; HGI—high glycemic index.
