# Supplementary material for: Administration of Hookworm Excretory/Secretory Proteins Improves Glucose Tolerance in a Mouse Model of Type 2 Diabetes
Source: Biomolecules. 2022 Apr 26;12(5):637. doi: 10.3390/biom12050637 (PMC9138508; doi:10.3390/biom12050637)
Supplement: Supplementary file 1 [file biomolecules-12-00637-s001.zip › biomolecules-1639916-supplementary.pdf]

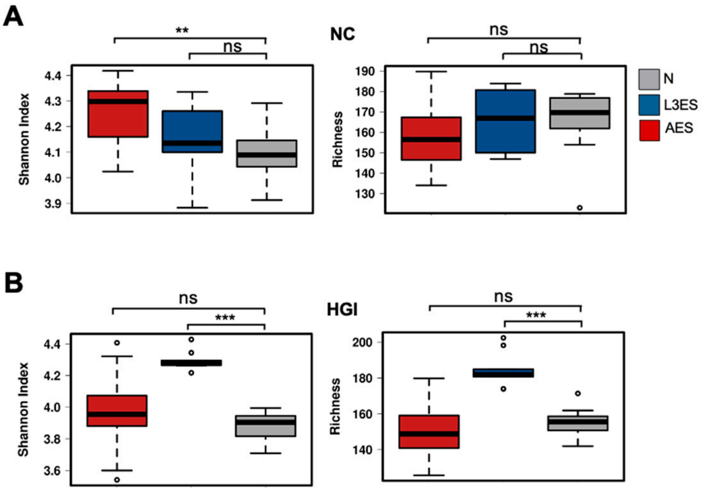

Supplemental Figure S1. Shannon index and richness in the small intestine of mice fed on NC diet or HGI diet and treated with crude *Nippostrongylus brasiliensis* L3ES or AES. *p* values are based on multiple linear regression and are from 2 pooled experiments where n = 5/group for each experiment. ** $p < 0.01$; *** $p < 0.001$.

Supplemental Table S1. Composition of diets fed to mice in this study. NC–normal chow; HGI–high glycemic index.

NC diet	HGI diet
barley	Casein (Acid) 200 g/Kg
Lupins	Dextrose 505 g/Kg
Soya meal	Canola Oil 50 g/Kg
Fish meal	Cocoa Butter 50 g/Kg
Mixed vegetable oils	Hydrogenated Vegetable Oil (Copha) 131 g/Kg
Canola oil	Cellulose 20 g/Kg
Wheat	
Salt	
Dicalcium phosphate	DL Methionine 3.0 g/Kg
Calcium carbonate	Calcium Carbonate 13.1 g/Kg
Magnesium oxide	Sodium Chloride 2.6 g/Kg
A Vitamin	AIN93 Trace Minerals 1.4 g/Kg
Trace mineral premix	Potassium Citrate 2.5 g/Kg
	Potassium Dihydrogen Phosphate 6.9 g/Kg
	Potassium Sulphate 1.6 g/Kg
	Choline Chloride (75%) 2.5 g/Kg
	AIN93 Vitamins 10 g/Kg
